# Medical and Philosophical Intelligence

**Published:** 1814-07

**Authors:** 


(To be continued.)

### MEDICAL AND PHILOSOPHICAL INTELLIGENCE.

ROYAL SOCIETY.—On Thursday, the 12th of May, a paper by Dr. BENJAMIN HAYNE on the Indian method of oxydizing silver by means of the juice of jatropha cureas, and on the milk of plants, was read. A piece of silver is heated to redness, wrapped in the the leaves of any kind of tree, and then quenched in the juice of the jatropha moluccana. This process is repeated about twenty times, taking care never to fuse the silver. The metal becomes quite brittle, and crumbles to powder between the fingers. Dr. Heyne tried the same process, substituting water instead of the vegetable juice, and a similar effect was produced. From Dr. Heyne's account of the above process, Dr. Thomson suspects that the silver is not oxydized, but merely rendered brittle, and reduced to a fine powder; probably by combining with something which exists in the vegetable juice employed, or rather in the cow-dung in which the silver is heated. The only known oxide of silver is a dark greenish brown powder, which is reduced to the metallic state by a very moderate heat. If the vegetable juice merely communicated oxygen, it is obvious that that principle would be driven off every time the metal was heated to redness, so that the process would never advance; but if the vegetable juice or cow-dung employed supplied sulphur, or any analogous principle, we can see how the repeated heatings would facilitate the combination, and how fusion would retard it.

---

*Linnean Society.*—On Tuesday, the 24th of May, the Anniversary Meeting of the Linnean Society of London was held at the Society's house in Gerrard-street, Soho, for the election of a Council and Officers for the present year, when the following members were elected of the Council, viz.

James Edward Smith, M.D.
Samuel, Lord Bishop of Carlisle.
Sir T. G. Cullum, Bart.
Philip Derbishire, Esq.
Mr. James Dickson.
Aylmer Bourke Lambert, Esq.
W. E. Leach, M.D.
Alexander Macleay, Esq.
Thomas Marsham, Esq.
Wm. George Maton, M.D.
Daniel Moore, Esq.
Joseph Sabine, Esq.
Thomas Smith, Esq.
William Smith Esq. M.P.
Edward Lord Stanley.

And the following were declared to be the Officers for the present year, viz.

James Edward Smith, M.D. President.

Samuel, Lord Bishop of Carlisle,

Wm. George Maton, M.D.

Thomas Marsham, Esq.

A. B. Lambert, Esq.

} Vice-Presidents.

Thomas Marsham, Esq. Treasurer.

Alexander Macleay, Esq.

Mr. Richard Taylor,

} Secretaries.

---

*Wernerian Society.*—On Saturday, the 5th of March, an interesting paper on the middle granite district of Galloway, was read by Dr. Grierson. It appears, from the doctor's observations, that this granite extends from eight to nine miles in one direction, and from three to four in another. It is coarse granular, sometimes porphyritic, but does not appear to be stratified. It is situate in the midst of distinctly stratified rocks, which on the east side of the

 granite granite mass dip easterly, on the west side westerly, or in both cases away from the granite; but on the north and south ends of the mass, the ends of the strata run directly towards it. The rock which rests immediately on the granite is a particular variety of compact fine granular gneiss, and cotemporaneous veins of the granite are to be observed shooting from the granite into this variety of gneiss. The gneiss seems to be connected with greywacke and greywacke slate, which are by far the most frequent of the stratified rocks in this tract of country. Limestone, hitherto a desideratum in the transition rocks of Galloway, was discovered by Dr. Grierson, in greywacke, near to Dalmellington; and it is highly probable that *workable* beds of limestone will be found among the stratified transition rocks of Galloway. The doctor also described several beds of felspar-porphyry, which he noticed in the greywacke of this part of Scotland.

At the following meeting, Professor Jameson gave an account of overlying primitive formations, as the first part of a dissertation on overlying rocks in general. From a series of observations which were made in the Highlands of Scotland, it appears that many of the primitive overlying sienite, granite, and porphyry formations of mineralogists, are not so in reality, but are thick conformable beds of these rocks which rise more or less above the surrounding strata. At the same meeting Professor Jameson described the Criffle district of granite and sienite, situate in the county of Galloway. These rocks occupy a considerable tract of country, and rise to the height of 1895 feet above the level of the sea; they are not distinctly stratified, and exhibit many interesting appearances, of apparent fragments, of cotemporaneous veins, and transitions into porphyry. The rocks which rest immediately on the granite or sienite are fine granular compact gneiss, slaty sienite, horneblende rock, and compact felspar rock. These rocks alternate with each other, and sometimes even with the sienite or granite; and cotemporaneous veins of granite are to be observed shooting from the granite into the adjacent stratified rocks. At the *Needle's Eye* on the west of Galloway, the Professor observed very fine examples of cotemporaneous veins and masses of granite, &c. in compact slaty felspar; and the felspar itself points out a hitherto-unsuspected connection of this mineral with certain kinds of clay-slate. On these rocks rest greywacke and greywacke slate, and sometimes transition porphyry; and it would appear, from Mr. Jameson's observations, that there is an almost uninterrupted transition from the gneissy rock into greywacke; and that when the felspar of the greywacke increases very much in quantity, becomes compact, dark-coloured, and slaty, the greywacke at length passes into clay-slate.

---

*Royal Medical Society, Edinburgh.*—The Royal Medical Society propose as the subject of their Prize Essay for the year 1815 the following question:

"The comparative specific caloric of veinous and arterial blood."

A set of books, or a medal of five guineas value, shall be given annually to the author of the best dissertation on an experimental subject proposed proposed by the Society; for which all the members, honorary, extraordinary, and ordinary, shall alone be invited as candidates.

The dissertations are to be written in English, French, or Latin, and to be delivered to the secretary on or before the 1st of December of the succeeding year to that in which the subjects are proposed, and the adjudication of the prize shall take place in the last week of February following.

To each dissertation shall be prefixed a motto; and this motto is to be written on the outside of a sealed packet containing the name and address of the author. No dissertation will be received with the author's name affixed; and all dissertations, except the successful one, shall be returned, if desired, with the sealed packet unopened.

---

*Kirwanian Society of Dublin.*—Dec. 1, 1813. A paper "On the crystallographical Method of Haüy," by Dr. J. O. Reardon, was read.

In this paper a concise statement of the theory of the learned Abbé was given, and also of the principal arguments brought forward in its support. The objections that have been offered to the system by various philosophers, as well as the replies of the Abbé, were then noticed; and a number of observations were made on the validity of the former, and the adequacy of the latter.

March 23, 1814. A paper "On an extensive Bed of Magnesian Limestone, found in the vicinity of Dublin," by S. Witter, Esq. was read.

An account of the analysis and of some peculiar circumstances attending the calcination of the stone, was first given. 36 per cent. of carbonate of magnesia were found in combination with 51 of carbonate of lime; the remaining portion being made up by silex, oxides of iron, and manganese. After some geological observations, the paper concluded with some remarks on the application of the mineral to the purposes of practical husbandry.

The same gentleman likewise read a series of observations, with an account of some experiments relating to the formation and properties of iodine. In allusion to the question of its elementary nature, he referred to some striking similarities in certain well-known compounds.

---

Two years ago Mr. Want discovered the composition of a medicine which possesses the power of removing the paroxysm of Gout in a degree fully equal to the Eau Medicinale. Since that period he has had abundant experience to satisfy himself of the identity of the two medicines.

The first hint he obtained on this subject was derived from the writings of Alexander of Tralles, a Greek physician of the sixth century, whose book on Gout is one of the most valuable clinical records of antiquity, and who, in his chapter on Anodynes, remarks, that some persons take a medicine called *Dia Hermodactylum,* which produces an evacuation of watery matter from the bowels, attended with such relief from pain that patients are immediately able to walk. *But,* says he, he, *it has this bad property, that it disposes them who take it to be more frequently attacked with the disease.** He speaks also of its producing nausea and loathing of food, and proceeds to describe the manner of counteracting its bad properties.—The effects here described are so similar to those resulting from the exhibition of the Eau Medicinale, that Mr. W. was led to hope that it might be the same medicine, or at least that it possessed powers of the same kind. The *Hermodactyl*, the basis of the composition, was strongly recommended by Paulus Ægineta as a specific for gout; and such was its reputation, that we are told by Quincy it had obtained the significant name of *Anima Articulorum*—the soul of the joints. He was further encouraged to think favourably of this medicine, from its having formed a leading article in the most celebrated gout-specifics of every age. Two of these are, Turner's gout powder, and the Vienna gout decoction, the latter of which is so strongly recommended by Behrens, in the *Ephemerides Naturæ Curiosorum*. It is likewise a fact notorious to every practitioner acquainted with the history of his profession, that this root has, at different periods, obtained considerable celebrity in the treatment of gout, though its general use has, after a time, been suspended; but that the occasional want of confidence in its powers arose less from its inefficiency than its misapplication, experience enables him to affirm.

The *hermodactyl* of the shops has been considered by most writers on the materia medica to be the root of the *Colchicum illyricum*; but some recorded accounts of the poisonous qualities of the *Colchicum autumnale*, and the manner in which death had been produced by it, induced Mr. W. to make his first trials with it, and his uniform success has rendered it unnecessary to make any change.

He directed a tincture to be made by infusing, for two or three days, a quantity of the fresh-sliced root of Colchicum autumnale, in proof spirits of wine, in the proportion of four ounces of the former to eight of the latter. This tincture he employed in all his first experiments, but as the efficacious parts of the plant are soluble in water or wine, either of these menstrua may be used; and, to produce a medicine more particularly resembling the Eau Medicinale in external circumstances, it is merely necessary to use good Sherry or Lisbon. Mr. W. purchased the root at Butler's, in Covent Garden, but it may be procured at all the physical herb shops, and under the vulgar name of *meadow saffron* may be found in every part of England.

The following is the prescription of the medicine referred to:—

Γραφὴ καθάρσιου του δι' ἑρμοδακτύλου ἀπλουστατη.—Ερμοδακτύλου δραχ. α. γιγγιβέρεως κε. θ. πεπέρεως κε. ϛ ἀνίσου κε. β. ταῦτα πάντα μία δόσις. ει δε θέλεις ἐπι πλέον ὑπάγειν την γαστέρα, προσμίγνυε σκαμμωνίας κε. δ. και ἀνύπως καθαίρει, καὶ ἀνωδύνους ποιει τοὺς πάσχοντας.

Alex. Trallian, cap. xi.—περι ανωδυνων αντιδοτων και φαρμακων καθαρτικων.

procured

For medicinal purposes, a recent infusion of the fresh or dried root in water is equally efficacious. Mr. W. has made extensive trials with this watery infusion, and never been disappointed in its effects. He was led to employ the dried root, from observing its variable strength when fresh, in which it appears to be much influenced by the weather and the season of the year. After rain, it contains a large quantity of water; but, on the contrary, after much sunny weather, the watery parts of the plant are evaporated, and the active qualities more condensed.

The dose of the tincture, whether it be made with water, wine, or spirit, should be the same, and should vary according to the constitution of the patient. Upon an average, we may fix two drams, or two ordinary tea-spoonfuls, as the proper quantity for an adult.

The wine of white Hellebore has been supposed by some to be the French medicine. At a very early period of the promulgation of this opinion, Mr. W. spared no pains to ascertain how far it was founded in fact. He has employed hellebore in every possible form. In some cases it appeared to be possessed of efficacy, but a series of disappointments induced him to abandon it as a medicine on which no dependence can be placed. In its mode of operation it has some properties in common with the Colchicum, or meadow saffron, but in its power of curing gout it falls infinitely short of it.

It is proper to state, that Mr. W.'s experiments have already been made in at least FORTY cases, followed by results of the most satisfactory nature, the paroxysms being always removed, and, in several instances, no return of disease having taken place after an interval of several months.

---

Mons. JUDELOT has published a recipe for a sulphurated soap, which he conceives to possess peculiar advantages and facilities in the cure of the itch. He prepares it, by dissolving six ounces of pulverized sulphuret of potass in a third of its weight of water. Two pounds of white soap are then to be rasped and put into an earthen vessel placed in a water bath; to which is to be added, gradually, two pounds of almond oil, triturating it well with the soap as it is added. The solution of sulphuret of potass is then to be put into a marble mortar, and well rubbed with the mixture of oil and soap, adding the latter very gradually. Two pounds of almond oil and two drachms of any agreeable essential oil are then to be added; and the mixture will be complete. This liniment should be kept in a close vessel, and used by rubbing an ounce of it upon different parts of the body, particularly those affected, twice a day. It rarely fails to cure the itch in eight days. It possesses the advantages of having no disagreeable odour, of not irritating the skin, and of preserving the patient's linen uninjured. It is cheap, and may be preserved for almost any length of time.—*Bulletin de la Faculté de Medicine.*

The same bulletin contains an account, by Mons. CHAUSSIER, of a case of small-pox, in which variolous pustules were found on the mucous membrane lining the trachea. The patient died apparently suffocated. The author concludes this notice by observing, that, in the same dissection, he was unable to inject the umbilical vein from the umbilical arteries; but mercury introduced into the umbilical vein passed after some lapse of time into the veins of the uterus. He attributes this not to a direct communication by continuity of canal between the fœtal and maternal veins, but to the transudation of the mercury through the membranous septum. He seems to think that the use of the umbilical arteries is the nourishment of the placenta.

---

Mons. Brisé Fradin has contrived an apparatus by means of which persons employed in atmospheres deteriorated by metallic or gaseous impurities may be prevented from breathing their noxious vapours. It consists in a cylindrical case of tin, open before, and fitted to a tube of glass at the upper extremity. The other end of the glass tube is to be introduced into the mouth, a little cotton placed in each nostril, and the tin cylinder, filled with moistened cotton, secured to the breast. Inspirations are then to be made through this apparatus, and expirations into the open air. Metallic vapours will be detained by the moistened cotton only, but if the noxious gases are to be avoided, it will be necessary to impregnate the cotton with some chemical agent, as ammonia, when chlorine is to be avoided. This contrivance appears adapted only to those cases when the exposure is of no long duration.

---

Mons. Chatelain has analysed a calculous concretion found in the jejunum of a man of sixty years of age. Contrary to the usual composition of biliary calculi, it contained no adipocire, nor yellow matter. It consisted of oxalate of lime and animal matter in the following proportions:

| | |
|---|---|
| Water - - - - - - - - - | ·10 |
| Inspissated bile - - - - - | ·02 |
| Green matter - - - - - - | ·08 |
| Oxalate of lime - - - - - | ·80 |
| | 1·00 |

---

We have observed with regret, in the French Journals lately received, the death of M. Le Gallois, the ingenious author of the Expériences sur le Principe de la Vie, an account of which we have concluded in the present Number of our Journal. We need not state how greatly physiology is indebted to the labours of this active experimentalist. A biographical memoir is announced in the Bulletin of the Faculty of Paris, which we hope soon to be able to lay before our readers.

---

Dr. Wigard, of Hamburgh, has discovered an excellent remedy in *Croup*. It consists in administering, according to the age and constitution, every hour, from two to three, or even from four to five grains of calomel, with the addition of half a grain, or at the most, one grain of moschus, to be continued till vomiting occurs. This vomiting happens in general after the use of the powders alone, and in most cases after the third dose; and (not, as has been asserted by most practitioners, a tough and white mucus, but) a substance of the consistency of cream of a greenish-yellow hue, is brought up, similar to to that which children bring up towards the latter stage of the hooping-cough, or such as is met with in the windpipe and bronchiæ of those children who have died of the angina. The earlier this vomiting sets in, the more certain and speedy is the cure. After this stage, Dr. W. ordered the powders to be given every two or two hours and a half, and a syrup of Oxym. Scillæ. Syrup. Senegæ, Ammonia Muriata, and Vin. Antimon. Huxh. from two to three tea-spoons full every hour or every hour and a half, in order to promote the vomiting and still more loosening the rising mucus.

---

A fœtus has been taken from the abdomen of a boy sixteen years of age, by Mr. Highmore, of Sherborne, Dorsetshire. The head and one of the lower extremities appear to have been removed by absorption, which process has commenced in some other parts. From the neck grows a quantity of hair twelve inches in length, and loose portions of it were found in the sac. The umbilical cord was connected with one part of the sac, there being no placenta. The patient during his illness was supposed to labour under diseased spleen, and died of hæmorrhagy from the stomach and bowels. We understand an account of the case, with engravings, will shortly be published. About six years ago, the account of a similar case was read to the Medico-Chirurgical Society, and has since been published in the first volume of their Transactions.

---

M. Bucholz has recently analyzed a new bitumen found in the environs of Halle in Saxony, which he thinks strongly resembles the *resino asphaltum* described by Mr. Hatchett some years ago in the Philosophical Transactions. According to M. Bucholz, that which is found at Halle is composed of two resins, one of which is very soluble in alcohol, and approaches to the vegetable resins, forming 91 parts of the bitumen; while the other, which forms nine parts, has some analogy to amber.

The following are a few of the most characteristic marks of this substance: It is found in balls the size of an apple, enveloped in gray crystallized gypsum; in colour it is brownish, or pale yellow; fracture glossy, and very brittle: it does not become soft under the fingers; it even does not melt so easily as other resins, but while melting it exhales an agreeable smell, something like that of animal resin and styrax. M. Bucholz remarks that, as Mr. Hatchett could dissolve only 55 parts of the bitumen examined by him, while the former dissolved 91, it is extremely probable that this difference was occasioned by Mr. Hatchett's using common alcohol.

The nine grains which were insoluble in alcohol were dissolved, but with much difficulty, in boiling oil. It was fusible in a strong heat, and gave out the smell of common resin.

The 91 parts above mentioned, when separated from the alcoholic solution, were dissolved by ether, and formed a brownish tincture, while ether of a specific gravity of 0·710, rectified over muriate of lime, made scarcely any impression. Oil of turpentine and rectified petroleum have little or no effect upon this resin. Caustic potash dissolved in two parts of water does not dissolve this resin; but, when the lixivium is decanted, the residue of the resinous principle is dissolved in water, from which we can separate the resin by the addition of muriatic acid.

---

Dr. Spurzheim will give a Course of Lectures on the Physiology of the Brain, or on the Organs by means of which the Faculties of the Mind manifest themselves, at his Rooms, No. 11, Rathbone-place, Oxford-street, on Monday the 11th of July next. He will comprise the whole in ten lectures, which will begin at two o'clock in the afternoon precisely, on Mondays, Wednesdays, and Fridays.

